# Intimate partner violence among women with multiple sclerosis

**DOI:** 10.1002/pcn5.70242

**Published:** 2025-11-11

**Authors:** Masoud Etemadifar, Omid Zamani, Anahita Bahrami‐Zadegan, Mehri Salari, Amir Reza Mansouri

**Affiliations:** ^1^ Department of Neurosurgery Alzahra University Hospital Isfahan University of Medical Sciences Isfahan Iran; ^2^ School of Medicine Isfahan University of Medical Sciences Isfahan Iran; ^3^ Faculty of Medicine Shahid Beheshti University of Medical Sciences Tehran Iran

**Keywords:** domestic violence, intimate partner violence, multiple sclerosis, women's health

## Abstract

**Aim:**

Intimate partner violence (IPV), a highly prevalent issue in women with chronic disorders, is rarely discussed and remains unrecognized, especially among patients with Multiple Sclerosis (MS). The objective of this study is to assess the prevalence and characteristics of IPV among female patients with MS.

**Methods:**

This cross‐sectional study was conducted from January to June 2024 among married women diagnosed with multiple sclerosis (MS) aged between 15 and 50 years, with a minimum duration of diagnosis of one year, in Isfahan MS center. IPV experiences were documented via a partially structured interview and a pre‐designed questionnaire. Chi‐square test, Fisher's exact test, and multivariate binary logistic regression were used to analyze the data.

**Results:**

Among 400 participants in this study, approximately 52% encountered incidences of IPV. Physical violence was the most prevalent form (60% of victims), followed by emotional (50% of victims) and sexual (16% of victims) forms. No significant association between incidents of IPV and demographic data of the patients and their spouses was found.

**Conclusion:**

Although IPV is considerably prevalent among female patients with MS, it severely lacks recognition. All women with MS, regardless of their sociodemographics, might be subject to violence. To prevent IPV, protective measures, including training programs for neurologists and neurology‐associated healthcare providers, must be employed. Future research should focus on exploring the negative effects of IPV on disease progression and prognosis.

## INTRODUCTION

The United Nations defines violence against women as *“Any act of gender‐based violence that results in, or is likely to result in, physical, sexual or psychological harm or suffering to women, including threats of such acts, coercion or arbitrary deprivation of liberty, whether occurring in public or in private life”*.^1^ Domestic violence (DV), defined as acts of violence against an intimate partner or spouse, is one of the most common forms of violence against women.^2^ Acts of domestic violence can be classified into three major groups: physical violence (physical behaviors that cause bodily harm), emotional violence (including harsh criticism, belittlement, and victim depersonalization), and sexual violence (any forceful attempt to engage in a sexual act).^3^, ^4^, ^5^ Intimate partner violence (IPV) is a subtype of DV perpetrated by a current or former intimate partner.^3^ IPV continues to be a significant concern among women, requiring substantial attention. World Health Organization (WHO) estimates that approximately one in three women worldwide has experienced IPV.^6^

Multiple sclerosis (MS), a chronic and debilitating immune‐mediated disorder of the central nervous system (CNS), impacts nearly three million lives globally. Even with current advances in achieving more effective treatments and continuous research efforts, MS still severely affects patients' quality of life. It is estimated that patients with MS lose approximately 13 quality‐adjusted life years (QALYs) per patient.^7^ The chronic and disabling course of MS increases patients' vulnerability to IPV, as individuals with chronic disabilities face higher risks of violence compared to able‐bodied populations.^8^ Moreover, MS disproportionally affects women, with incidence rates two to three times higher than in men.^9^, ^10^ Therefore, IPV among women with MS warrants urgent attention.

Despite the critical importance of this issue, data on the subject remains extremely limited. This study aims to assess the prevalence and risk factors of IPV among women with MS.

## METHODS

### Study design, setting, and participants

The reporting of this study was in accordance with the guidelines outlined in the STROBE (Strengthening the Reporting of Observational Studies in Epidemiology) statement.^11^ This is a descriptive, observational, cross‐sectional study, conducted from January 2024 to June 2024, among patients with MS admitted to the Isfahan Multiple Sclerosis Center (IMSC), Isfahan, Iran.

Utilizing the census method, married women aged 15 to 50 years, who had been diagnosed with MS for at least one year, were included in the study. The diagnosis was done by a neurologist based on the 2017 revision of the McDonald criteria for MS.^12^ Patients who met any of the following criteria were excluded: i) Age outside the specified range (over 50 or under 15 years); ii) Diagnosis of less than one year; iii) Single, widowed, or divorced women; iv) History of domestic violence prior to MS diagnosis; v) Presence of any concurrent physical disabilities or established psychological disorders.

### Data collection, variables, and ethics

Data were collected through a partially structured interview and a pre‐designed questionnaire, both prepared and performed in Persian. The pre‐designed questionnaire was previously developed by Mohseni Tabrizi et. al. and confirmed for validity and reliability.^13^ The reliability of the questionnaire for this study was confirmed, with a Cronbach's alpha of 0.77. The interviews were carried out by a physician in a private room, ensuring confidentiality by excluding the participants' husbands. Each interview lasted approximately 30 to 45 min. The studied variables included demographic information of the participants and their husbands (age, duration of marriage, education, and husbands' substance addiction status), as well as IPV status assessment, including its type and frequency. This study adheres to the principles of the World Medical Association Declaration of Helsinki. Ethical approval of this study was obtained from the ethics committee of Isfahan University of Medical Sciences (Approval ID: IR.MUI.MED.REC.1399.920). Written informed consent was acquired from the participants prior to their inclusion in the study.

### Statistical analysis

Mean and standard deviation (±SD) were used to describe the continuous variables, while frequency and percentage (%) were used for the categorical and qualitative data. Chi‐square and Fisher's exact tests were used to analyze and compare qualitative variables. A multivariate binary logistic regression analysis was also conducted. A *p*‐value of less than 0.05 was considered statistically significant. All data were described and analyzed using the IBM SPSS software (version 27.0 for Windows 10).

## RESULTS

In this study, 400 married women with MS aged 15 to 50 years were interviewed. Table 1 summarizes the demographic information of the studied patients and their husbands.

**Table 1:** Demographic information of the patients and their husbands.

Demographic information of the patients	
Mean (±SD) age/Years	35.00 (±7.80)
Mean (±SD) duration of MS/Years	8.20 (±6.07)
Mean (±SD) Duration of marriage/Years	10.92 (±7.76)
**Demographic information of the patients’ husbands**	
Mean (±SD) age/Years	39.85 (±7.99)
Level of education/Frequency (percentage)	
No formal education	1 (0.3)
Primary education	154 (38.5)
Secondary education	234 (58.5)
Tertiary education	11 (2.8)
Addiction status/Frequency (percentage)	
None	314 (78.5)
Alcohol	10 (2.5)
Cigarettes	50 (12.5)
Illicit drugs	26 (6.5)

Abbreviations: MS, multiple sclerosis; SD, standard deviation.

Based on the interviewed data, more than half of the patients (207, 51.75%) experienced at least one form of IPV. Among the three types of violence, physical violence was the most prevalent form (approximately 31% of the patient population), followed by emotional (27.5% of the population) and sexual (approximately 9% of the population) violence. Assault (slapping, beating, kicking), financial arguments, threats, and sexual coercion were the most common forms of physical, emotional, and sexual violence, respectively. Fifty‐six patients experienced more than one form of violence, and eight patients (2%) suffered from all forms. Approximately half of the victims (100, 48.5%) were occasionally (once a month or less) exposed to IPV, while 25 patients (6.2%) reported experiencing daily violent incidents. Table 2 presents the distribution of various types of IPV.

**Table 2:** Distribution of different forms of intimate partner violence.

Type	Frequency (percentage)
Physical	125 (60.38)
Assault	71 (34.30)
Kicking	38 (18.36)
Beating/slapping	33 (15.94)
Object throwing	29 (14.00)
Others	25 (12.08)
Emotional	110 (53.14)
Financial coercion	33 (15.94)
Vilification	30 (14.49)
Humiliation/belittling	24 (11.59)
Abandonment threats	23 (11.11)
Sexual	35 (16.90)
Sexual coercion	18 (8.69)
Sexual assault	17 (8.21)
Total	207

Subgroup analysis of acquired data from patients and their husbands and the results of multivariate logistic regression analysis showed no significant relationship between demographic and socioeconomic status (e.g., age, duration of marriage, education, addiction) and the prevalence of experienced IPV (Table 3). The frequency of reported violent behaviors was non‐significantly greater among husbands with higher levels of education. Additionally, a non‐significant trend was observed with the prevalence of IPV increasing with the patients' age. Moreover, no association was observed between the duration of MS and IPV prevalence.

**Table 3:** Association between sociodemographic characteristics of patients and their husbands and the prevalence of intimate partner violence.

Characteristics	Experience of domestic violence		Statistical values
	No (percentage)	Yes (percentage)	
Patients' age group (in years)			
18–25	29 (51.8)	27 (48.2)	*P* = *0.322*
25–35	67 (48.6)	71 (51.4)	
35–45	84 (50)	84 (50)	
>45	13 (34.2)	25 (65.8)	
Husbands' level of education			
No education	1 (100)	0 (0)	*P* = *0.129*
Primary	85 (53.9)	71 (46.1)	
Secondary	107 (45.7)	133 (55.4)	
Tertiary	2 (18.2)	9 (81.8)	
Duration of marriage (years)			
<5	67 (50.8)	65 (49.2)	*P* = *0.778*
5–10	35 (46.7)	40 (53.3)	
>10	91 (47.2)	102 (52.8)	
Husbands' addiction status			
Alcohol	7 (70)	3 (30)	*P* = *0.168*
Cigarette	29 (58)	21 (42)	
Drugs	14 (53.8)	12 (46.2)	
Husbands' age group (years)			
<25	2 (28.6)	5 (71.4)	
25–30	33 (52.4)	30 (47.6)	*P* = *0.287*
30–35	13 (36.1)	23 (63.9)	
>35	145 (49.3)	149 (50.7)	
Duration of MS (years)			
<5	79 (46.7)	90 (53.3)	*P* = *0.084*
5–10	58 (57.4)	43 (42.6)	
>10	56 (43.1)	74 (56.9)	

Abbreviation: MS, multiple sclerosis.

## DISCUSSION

This study was among the very limited number of studies to investigate intimate partner violence (IPV) among women with MS. According to the results, more than half of the women with MS reportedly experienced IPV during the course of their disease. The most prevalent forms of IPV were physical, verbal/emotional, and sexual violence, respectively. Furthermore, violence against women with MS is possibly unrelated to the demographic characteristics of the patients and their spouses, as indicated by this study.

Demonstrably, the prevalence of IPV against women with MS is high, corroborated by the finding of a previous study by Manuchehri et al.^14^ Evidently, a majority of patients with MS are also subjected to DV and neglect by other family members and/or close friends.^15^ Several factors contribute to the high prevalence of DV and IPV among patients with MS. Physical disabilities significantly increase the risk and frequency of violence.^16^ Age at onset of MS peaks in the ages between 30 and 45,^17^ which coincides with the age group most frequently associated with police‐reported cases of IPV.^18^ MS affects multiple aspects of life for both patients and their caregivers, including quality of life, interpersonal intimacy, spousal relationships, and overall life satisfaction.^19^, ^20^ It also damages patients' economic and social status, leading to financial dependency,^21^ which is a major risk factor for violence.^22^ According to a previous study, there is a negative correlation between the overall rate of IPV and economic factors, including income.^23^ Moreover, physical capacity and work capability are severely affected among patients with MS,^24^ further precipitating their financial decline. Furthermore, treatment of MS places a heavy economic burden, especially in the Middle East, where the cost of treatment per patient exceeds nominal gross domestic product (GDP) per capita in some regions.^25^ Approximately 30% of individuals with MS require specialized domiciliary support, most of which is provided by their spouses.^21^, ^24^ Dependency on spouses for several daily life aspects, in addition to the caregiving burden, may play a role in the dynamics of violent behaviors.

Contrary to the findings from previous studies, physical violence was the most prevalent form among women with MS in this study.^14^ Intriguingly, sexual violence was the least frequently reported form of DV across all regional studies, including this one.^14^, ^26^ This could be due to underreporting of such violence, which might stem from several reasons, including patients' hesitation to answer questions about their sexual lives and experiences of violence, the perception that such questions are “too personal,” and varying interpretations of what constitutes sexual violence. Additionally, stigma and social shame surrounding victims may further lead to sexual violence being rarely addressed or openly discussed.^27^ Moreover, socio‐cultural norms and legal framework in Iran might be influential in such disparities.

IPV was unrelated to the sociodemographic status of patients and their spouses in this study. This implies that clinicians should be trained to recognize signs of abuse and have referral pathways to appropriate support services.

By educating patients about IPV, its potential impact on disease progression, and their rights regarding this matter, patients may feel empowered to disclose instances of violence openly.^28^ Most women disclose IPV incidents to family members, which might result in negative reactions, leading to a feeling of re‐victimization, negatively affecting their mental health.^29^ On the other hand, seeking help from social groups and receiving positive reactions may boost patients' mental health.^30^ Primary violence preventive measures, including life skills development, as well as secondary preventive strategies, including screening tools and educational programs for healthcare professionals, can be highly effective.^31^ According to a previous study, women's support from husbands' families is an IPV preventive factor.^22^ Thus, performing informative sessions for husbands' close families regarding MS and its associated difficulties might be a logical strategy. Depression and anxiety are prevalent among people with MS, and over time, patients may become increasingly isolated from their social network.^24^, ^32^ IPV incidents can further deteriorate patients' psychological state. Specialized psychological support interventions are recommended for MS patients who have suffered from IPV.

This was the first study conducted specifically among women with MS in Isfahan, Iran. Similar studies should be conducted in other regions of Iran and the Middle East to provide a more comprehensive picture of violence among MS patients and individuals with other medical conditions. Moreover, little is known about the relationship between DV and clinical course and outcomes of MS, highlighting the critical need for investigation in this matter. Neurologists and other healthcare providers working in neurology clinics and hospitals are prompted to notice IPV among women with MS and take appropriate preventive and supportive measures.

## LIMITATIONS

It is important to acknowledge several limitations in this study that might affect the results. The single‐center design limits the generalizability of the study's findings. Data was collected using self‐report instruments, which might cause several types of biases, including the recall and social desirability bias. Additionally, this study mainly focused on IPV. However, there are other instances of DV perpetrated by other close family members, which require further investigation. Moreover, male patients might also suffer from domestic violence, which remains an overlooked matter.

## CONCLUSION

Intimate partner violence (IPV) is a prevalent yet overlooked issue among women with MS. Despite its significant impact, IPV remains unrecognized in MS care programs. Any woman with MS is at risk of violent behaviors, regardless of their socioeconomic status. Specialized support programs for IPV are essential. Training clinicians to recognize the increasing risk of IPV and the importance of regular monitoring on this matter is prompted. Future research should focus on developing effective protective measures and evaluating the impact of IPV on disease progression and clinical outcomes in MS.

## AUTHOR CONTRIBUTIONS

**Masoud Etemadifar**: conceptualization, methodology, project administration, supervision. **Omid Zamani**: methodology, formal analysis, writing‐original draft, project administration. **Anahita Bahrami‐Zadegan**: writing—original draft, writing—review and editing. **Mehri Salari**: conceptualization, writing—review and editing, supervision. **Amir Reza Mansouri**: methodology, formal analysis, writing—original draft, writing—review and editing.

## CONFLICT OF INTEREST STATEMENT

The authors declare no conflicts of interest.

## ETHICS APPROVAL STATEMENT

Ethical approval of this study was obtained from the ethics committee of Isfahan University of Medical Sciences (Approval ID: IR.MUI.MED.REC.1399.920).

## PATIENT CONSENT STATEMENT

Written informed consent was acquired from the participants prior to their inclusion in the study.

## CLINICAL TRIAL REGISTRATION

N/A.

## Data Availability

The data that support the findings of this study are available from the corresponding author upon reasonable request.
